# Shed light on photosynthetic organisms: a physical perspective to correct light measurements

**DOI:** 10.1007/s11120-023-01001-5

**Published:** 2023-02-23

**Authors:** Andreas Walter, Harald Schöbel

**Affiliations:** Department of Biotechnology & Food Engineering, MCI—The Entrepreneurial School, Maximilianstraße 2, 6020 Innsbruck, Austria

**Keywords:** LED, Illumination, Irradiation, Photosynthesis, Vertical farming, Algae

## Abstract

The requirements for novel and innovative production systems expedite research on light emitting diode-based illumination in a life science context. In course of these rapid developments, the scientific community is in need of a consensus regarding to the characterization and presentation of the applied lighting conditions. This publication aims to establish a basic understanding of photon physics and propose guidelines for the conclusive usage of light related quantities. To illustrate the challenges in data handling, six different light sources were measured and characterized. Furthermore, a stepwise conversion within and in-between physical systems is presented, and an opportunity to extract information from weak data sets is demonstrated. The proposed calculations indicated flexibility in data handling, but revealed partial inaccuracy for colored light emitting diodes with spectral power distribution maxima far-off 550 nm compared to spectrometer-based measurements and conversions. Furthermore, it could be shown, that when comparing light properties, the determination of photometric quantities is incorrect to describe lighting systems for photosynthetic organism and the usage of luxmeter or similar photometric sensors should be avoided. The presented guidelines shall support scientists in applying a consistent and precise characterization of their illumination regimes, tailored to their requirements to avoid ambiguous communication and the generation of incorrect and thus incomparable data based on wrong quantities and units, such as lumen or lux, in future research.

## Current status in bio-illumination

Light is the essential source for photosynthetic activity (Singh and Singh 2015) and its quality (color, wavelengths), quantity (involved energy, number of photons) and photoperiod (duration of illumination) regulates growth, development and productivity in phototrophic organisms (Folta and Childers 2008). Sunlight bears the advantage of suppling a free and abundant light source (Blanken et al. 2013), however a rising world’s population—demands for approximately nine billion inhabitants have to be covered in 2050—and an increasingly challenging global environment, reinforce the needs for novel and innovative production systems, such as vertical farming based on hydro-, aqua- or aeroponics (Darko et al. 2014).

Most of these controlled growing facilities require, at least partially, artificial light (Darko et al. 2014): Fluorescent lamps, particularly those having enhanced blue and red spectra (Blanken et al. 2013) and high intensity discharge lamps, such as metal halide and high pressure sodium lamps are still widely used in greenhouses and growth chambers (Blanken et al. 2013; Darko et al. 2014). However, broad light spectra and difficult to control intensities of fluorescent lights, as well as high operational temperatures for high intensity discharge lamps, strongly limit their application in innovative production systems (Darko et al. 2014). Light emitting diode (LED) based technologies are in the first place to substitute these light sources and to revolutionize controlled growing systems: LED emit almost monochromatic light with a narrow spectral distribution or white light within a wide range of correlated color temperature from 2700 K (warm light) to more than 6000 K (cold light) (Baidya et al. 2021; Muñoz-Fernández et al. 2021), which allow the design of well-defined, species-specific illumination or irradiation conditions that may be adjusted throughout the species’ life and production cycles (Folta and Childers 2008). Their compact layout enables an easily integration into experimental setups and by using additional optics like lenses or reflectors, unique light regimes can be achieved. In addition, they are environmentally friendly, due to their long durability, their energy efficiency and their lack of toxic elements such as mercury (Lima et al. 2021). Due to these advantages as well as the easy availability of LED-based illumination systems, their utilization in photobiology and other life-science areas is emerging. LED technologies are used among others for UV disinfection purposes (Song et al. 2016; Li et al. 2017) or in photomedicine, like dental applications (Wiggins et al. 2004), in dermatology (Jagdeo et al. 2018) or for photodynamic therapy (Hempstead et al. 2015; Fiala et al. 2021). Regarding biomass and metabolite production in plants (Bantis et al. 2018; Ju et al. 2022), microalgae (Moreira et al. 2021; Mutschlechner et al. 2022) and cyanobacteria (Oren et al. 2021) artificial lighting based on LED technology is to be on the rise.

Thus, research and development of LED-based technologies is on the drive. Their spreading application for bio-illumination can be reflected by total numbers of respective publications: A literature research on “light emitting diode” and “biology” on google scholar (29.09.2022) revealed an almost exponential gain where the absolute numbers of publication per year increased by a factor of three in the last ten years (3720 in 2011 vs. 11.500 in 2021). While the use of illumination technologies in the biological context is undisputed an emerging research field, parts of the scientific community seems to lack a consensus, when it comes to the characterisation and presentation of their applied illumination or irradiation conditions: According to a literature research, on google scholar (01/17/2023), 4650 publications (2020–2023) used “lux” or “lx”—a unit based on the sensitivity of the human eye and considering the involved energy—and 3035 “μmol m^−2^ s^−1^” (and seven other variations: "µmolm-2 s-1", "µmol m-2 s-1", "µmolm-2 s-1", "µmols-1 m-2", "µmol s-1 m-2", "µmols-1 m-2" and "µmol s-1 m-2")—a unweighted unit considering the involved light particles (photons)—when describing illumination conditions for different “green algae”. As there is no correlation between the human visual perception and the light response of green algae, this literature search suggest that almost two third of the publications used improper quantities to describe light related conditions, thus underlining the need for a harmonization in “terminology”.

This publication introduces the most common physical systems to describe photo-illumination—namely, energy- and photon-based systems—and shed light on the use of biological weighting functions (BWF). To point out potential pitfalls when comparing light data from literature or using manufacturer datasheets, light related quantities of six artificial light sources were investigated based on their power output and spectral properties and characterized using different weighted and unweighted systems. To be more precisely, irradiance and spectral power distribution of a cold white fluorescent lamp, a neutral white LED and four different single colored LEDs were measured, compared and, thereafter, converted within the energy-based system from radiometric to photometric systems and vice versa as well as from the energy- to the photon-based system. Furthermore, a quick approximation method to convert light related data between common used quantities without sophisticated calculation will be introduced and a guideline to measure light regimes with proper sensors will be given Our findings shall support scientist to apply a consistent and precise characterization of their illumination regimes, tailored to their requirements in order to avoid ambiguous communication and the generation of incomparable data in future research.

## Introducing the most common physical systems to describe photo-illumination

To quantify photo-illumination, various systems have evolved over time. Depending on the field of application, the involved total energy per time or the number of present photons were decisive to characterize lighting conditions. Hence, energy-based systems or photon-based systems were introduced. Biological weighting functions were additionally applied, when the response from the illuminated system had to be taken into account. In the following section, common weighting functions and the related physical quantities to describe photo-illuminant were discussed.

### Biological weighting functions

**Fig. 1:**
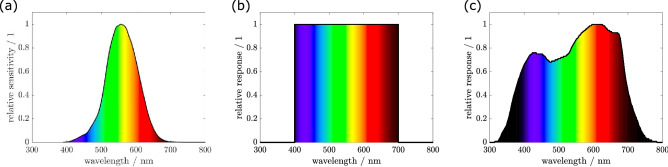
Common biological weighting functions. **a** The human visual perception quantifies the wavelength response of the human eye, by taking the spectral sensitivity of the human visual perception of brightness into account. **b** The photosynthetically active radiation designates the spectral range in which photosynthetic active organisms are capable to perform the process of photosynthesis. **c** The average plant response, also known as the McCree Curve (McCree 1972) or the Plant Sensitivity Curve, represents the average photosynthetic response of plants to light energy. Figures based on data from (Sager et al. 1988)

To fully describe the impact of light on biological systems, empirical relationships are in need to quantify the effectiveness of light (= relative response) on target organisms relative to the applied wavelengths (Andreasson and Wängberg 2006). The “weight” of these effects can be described in terms of biological weighting functions. Common weighting functions are the luminous efficiency function (Pirenne 1962), representing the average spectral sensitivity of human visual perception of light from 380 to 800 nm, the photosynthetically active radiation (PAR) (McCree 1981), which designates the spectral range from 400 to 700 nm or the average plant response, from 360 to 760 nm (McCree 1972), which considers the efficiency regarding photosynthesis (Fig. 1). By convoluting the biological weighting function with the spectral power distribution of the light source, the impact of the illumination can be described specific to the involved biological system.

### Energy-based systems

Energy-based systems, predominantly described via radiometric and photometric quantities, reflect the emitted energy from a light source or the incident energy on a certain area of interest and various quantities are in use (Meyer-Arendt 1968). Both, radiometric and photometric quantities, describe akin characteristics such as emitted flux or intensity, however, they differ regarding measurement standard and units as well as relevant spectral ranges: Radiometric quantities are absolute and can be used for the whole electromagnetic spectrum. Photometric quantities consider the wavelength dependent sensitivity of the human eye and are limited to the visible region of the electromagnetic spectrum, which is approximately from 380 to 800 nm.

An overview of radiometric and photometric quantities mentioned in this work is stated in Table 1. This overview is by no means exhaustive, however, should cover the most important quantities, used to describe light-based experiments for life-science approaches. To distinguish between radiometric and photometric quantities in this work, all their symbols were indexed with an additional $R$ or $P$ for radiometric or photometric quantities, respectively (e. g. radiant flux ${\Phi }_{R}$ versus luminous flux ${\Phi }_{P}$).

**Table 1:** Overview of basic radiometric and photometric quantities for life-science applications.

simplified definition	name	unit
emitted energy $Q$	radiant energy ${Q}_{R}$	joule $(\mathrm{J})$ or watt second $(\mathrm{W}\bullet \mathrm{s}$)
	luminous energy ${Q}_{P}$	lumen second $(\mathrm{lm}\bullet \mathrm{s})$
emitted energy per second or flux or power $\Phi =\frac{Q}{t}$	radiant flux ${\Phi }_{R}$	watt $(\mathrm{W})$ or joule per second $(\mathrm{J}\bullet {\mathrm{s}}^{-1})$
	luminous flux ${\Phi }_{P}$	lumen $(\mathrm{lm})$
emitted flux per solid angle $I=\frac{\Phi }{\Omega }$	radiant intensity ${I}_{R}$	watt per steradian $(\mathrm{W}\bullet {\mathrm{sr}}^{-1})$
	luminous intensity ${I}_{p}$	candela $(\mathrm{cd})$ or lumen per steradian $(\mathrm{lm}\bullet {\mathrm{sr}}^{-1})$
incident flux on a surface $E=\frac{\Phi }{A}$	irradiance ${E}_{R}$	watt per square meter $(\mathrm{W}\bullet {\mathrm{m}}^{-2})$
	illuminance ${E}_{P}$	lux $(\mathrm{lx})$ or lumen per square meter $\left(\mathrm{lm}\bullet {\mathrm{m}}^{-2}\right)$
emitted flux from a surface per solid angle $\frac{\Phi }{A\bullet\Theta }$	radiance ${L}_{R}$	watt per square meter per steradian ($\mathrm{W}\bullet {\mathrm{m}}^{-2}\bullet \mathrm{sr} )$
	luminance ${L}_{P}$	candela per square meter $\left(\mathrm{cd}\bullet {\mathrm{m}}^{-2}\right)$ or lumen per square meter per steradian ($\mathrm{lm}\bullet {\mathrm{m}}^{-2}\bullet \mathrm{sr})$

Next to a simple definition of each quantity, the particular name of each quantity in the radiometric (unweighted) and the photometric system (weighted with the luminous efficiency curve) including their SI unit are summarized

**Fig. 2:**
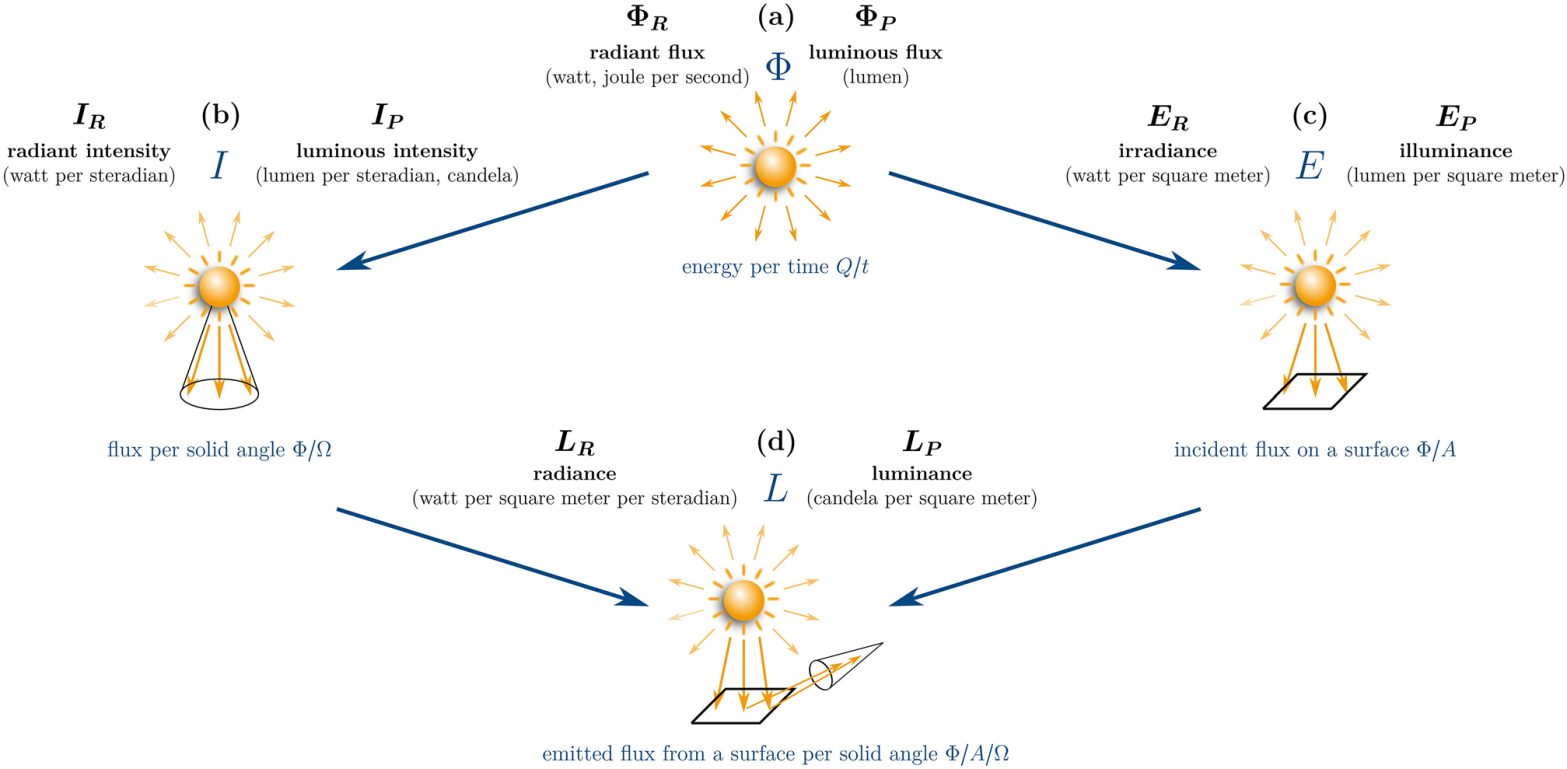
Schematic illustration of radiometric and photometric quantities. **a** A light source emits a certain amount of energy per time $Q/t$. This is commonly referred to as emitted power or flux $\Phi ,$ more precisely radiant flux ${\Phi }_{R}$ (radiometric system) or luminous flux ${\Phi }_{P}$ (photometric system). Depending on the classification, a distinction between direction **b** or incident surface **c** can be made. Considering the emission direction, the flux per solid angle $I=\Phi /\Omega$ is the relevant quantity **b**, which is termed radiant intensity ${I}_{R}$ (radiometric system) or luminous intensity ${I}_{P}$ (photometric system). To describe the transported energy per time onto a certain surface, the incident flux on a surface $E=\Phi /A$ is used **c**, which is referred to as irradiance ${E}_{R}$ (radiometric system) or illuminance ${E}_{P}$ (photometric system). Combining area size and direction in space, the flux emitted, reflected or transmitted from a certain area into a certain solid angle $L=\Phi /\mathrm{A}/\Omega$ comes into effect **d**. This quantity is called radiance ${L}_{R}$ (radiometric system) or luminance ${L}_{P}$ (photometric system)

Each light source radiates a certain amount of energy $Q$ per time $t$, also called radiant flux ${\Phi }_{R}$. The radiant flux, referred to as radiant power as well, is a radiometric quantity with the unit watt or joule per second. The corresponding photometric quantity would be the luminous flux ${\Phi }_{P}$ or luminous power in lumen. From the flux $\Phi$, many other quantities can be derived. Figure 2 shows a schematic illustration of common light related quantities and visualize their correlation.

Taking the spatial distribution of the emitted light into account, the radiant intensity ${I}_{R}$ in watt per steradian (sr) and the luminous intensity ${I}_{P}$ in candela (cd) are used. The unit candela is defined as the emitted lumen per steradian. These quantities describe how much energy per time is emitted into a certain direction in space and are defined as the amount of radiated flux into a certain solid angle $\Omega$. A solid angle is given in steradian $(\mathrm{sr})$ and can be seen as a three-dimensional analogy to the two dimensional radian measure of an angle, meaning for example the radian measure of a complete circle is $2\pi \mathrm{rad}$ (circumference of the unit circle) and the solid angle of a complete sphere is $4\pi \mathrm{sr}$ (surface area of the unit sphere). Isotropic light sources, such as the sun or in good approximation a light bulb, emit homogeneous in all spatial directions. In contrast, LEDs radiate according to the Lambert’s cosine law only in the front half-space with a certain viewing angle. Taking the surface of the incident light into account, the irradiance ${E}_{P}$ in watt per square meter or the illuminance ${E}_{P}$ in lux or lumen per square meter are used. Irradiance and illuminance represent the incident flux on a given surface area $A$. These quantities are the primary quantities that are measured with light measurement devices, commonly referred to as radiometer or luxmeter. From a formal point of view, the irradiance can also be called intensity as it represents the transferred power per unit area. The radiance ${L}_{R}$ in watt per steradian per square meter and the luminance ${L}_{P}$ in candela per square meter describes the emitted radiant flux by a surface into a certain solid angle. More precisely, the considered light from the surface can be emitted, reflected or transmitted by the surface into a certain direction. As this quantity takes the size of the light source into account, it is useful to evaluate the impact of the source regarding perception and is an indicator for subjectively perceived brightness. A too high radiance or luminance can lead to disturbing glare effects. The luminance is often given in the specification of computer screens or cellphone displays to quantify the brightness. The intensity (irradiance/luminance) is a favorable quantity to characterize lighting systems, as it reflects the incident energy per time and area on the biological system best.

### Photon-based system

Photon-based quantities reflect the particle character of light, where light is pictured as light quanta or photons and describe the number of emitted photons from a light source or the number of incident photons on a certain area. The number of photons $N$ corelates with the total involved energy $Q$ and the individual energy of a single photon ${Q}_{\mathrm{photon}}$. As the energy of a single photon depends on the wavelength

1 $${Q}_{\mathrm{photon}}=h\bullet \frac{c}{\lambda },$$

where $h={6.626\bullet 10}^{-34} \mathrm{J s}$ is the Planck constant and $c=299 792458 \mathrm{m }{\mathrm{s}}^{-1}$ is the speed of light, the number of photons can be calculated from

2 $$N=\frac{Q}{{Q}_{\mathrm{photon}}}=\frac{Q\bullet \lambda }{h\bullet c}$$

With this basic relation first described by Albert Einstein (Einstein 1905), all related photon-based quantities can be derived. In life-sciences the photon flux density ($$PFD$$) in micromole per second and per square meter ($$\mathrm{\mu mol }{\mathrm{s}}^{-1} {\mathrm{m}}^{-2}$$) is an adequate quantity in a photon-based system, sometimes also termed as quantum flux density (McCree 1973). This quantity specifies the number of incident photons per seconds on an unit area as multiple of the Avogadro constant $${N}_{A}=6.022\bullet {10}^{23} {\mathrm{mol}}^{-1}$$. Occasionally the unit microeinstein per second and per square meter ($$\mathrm{\mu E }{\mathrm{s}}^{-1} {\mathrm{m}}^{-2}$$) is used, which is no SI unit but describes the same. The $$PFD$$ is an unweighted quantity. In the context of photosynthesis, the $$PFD$$ is additional weighted with the PAR function (Fig. 1b) resulting in photosynthetic photon flux density $$(PPFD)$$ or weighted with the plant sensitivity curve (Fig. 1c) resulting in the yield photon flux density ($$YPFD)$$. Integrating these quantities over the incident area, the resulting quantities are photon flux $$(PF$$; unweighted), photosynthetic photon flux $$(PPF$$) and yield photon flux $$(YPF$$). An overview over common photon-based quantities is stated in Table 2. A further integration over time would lead to the total number of involved photons.

**Table 2:** Overview of basic quantities in a photon-based system used for life science applications.

simplified definition	name	unit
emitted/incident number of photons	number photons $(N)$	micromol $(\mathrm{\mu mol})$ or microeinstein $(\mathrm{\mu E})$
emitted/incident number of photons per second	photon flux $(PF)$	micromol per second $(\mathrm{\mu mol}\bullet {\mathrm{s}}^{-1})$ or microeinstein per second $(\mathrm{\mu E}\bullet {\mathrm{s}}^{-1})$
	photosynthetic photon flux $(PPF)$	
	yield photon flux $(YPF)$	
emitted/incident number of photons per area and per second	photon flux density $(PFD)$	micromol per square meter per second $(\mathrm{\mu mol}\bullet {\mathrm{m}}^{2}\bullet {\mathrm{s}}^{-1})$ or microeinstein per square meter per second $(\mathrm{\mu E}\bullet {\mathrm{m}}^{2}\bullet {\mathrm{s}}^{-1})$
	photosynthetic photon flux density $(PPFD)$	
	yield photon flux density $(YPFD)$	

Next to a simple definition of each quantity, the particular name of each quantity in a unweighted and a weighted system (weighted with the BWF PAR) and weighted with the average plant response curve (McCree 1971) including their SI units are summarized

## Material and methods

Six different light sources were used in this study: Four single colored LEDs—amber, green, red, violet—(Luxeon CZ color line, Lumileds), a neutral white fluorescent lamp (8TLD18W/865, Philips) and a neutral white LED (SZ55-MO-WO-C8, SEOUL Semiconductor). The spectral power distribution, used for converting and comparing different light related quantities, was measured with a spectrometer MAYA 2000 Pro equipped with a diffraction grating #HC-1 and an entrance slit of 5 µm (Ocean Insights, Rostock, GER), resulting in a spectral resolution of 0.66 nm FWHM. Light was coupled into the spectrometer via an optical fiber with a core diameter of 600 µm (QP600-1-SR-BX, Ocean Insights) and a cosine corrector (CC-3-UV-S, Ocean Insights). The spectrometer was calibrated with a wavelength calibration source (mercury-argon HG-2, Ocean Insights, Rostock). The conversion of radiometric quantities into photometric quantities, and vice versa, as well as the calculation of the $PFD$ were performed with MATLAB 2021b (The Mathworks 2021).

## Results and discussion

### Comparing light sources in energy-based systems

In the energy-based system, common used quantities are unweighted radiometric quantities and photometric quantities, which are radiometric quantities weighted with the luminous efficiency curve $V\left(\lambda \right).$ Hence, if the spectral power distribution of a certain light source is available, a conversion from one system into the other is possible. Starting from a known spectral power distribution ${\Phi }_{R}\left(\lambda \right)$ the total luminous flux (${\Phi }_{P};\mathrm{in lumen})$ can be calculated with the Eqs. (3a) or (3b),

3a $${\Phi }_{P}={K}_{m}\bullet \underset{380 \mathrm{nm}}{\overset{800 \mathrm{nm}}{\int }}{\Phi }_{R}\left(\lambda \right)\bullet V\left(\lambda \right)d\lambda$$

3b $${{\Phi }_{P}=K}_{m}\bullet \sum_{380 \mathrm{nm}}^{800 \mathrm{nm}}{\Phi }_{R}\left(\lambda \right)\bullet V(\lambda ),$$

where ${K}_{m}=683\frac{\mathrm{lm}}{\mathrm{W}}$ is the maximal luminous efficacy, which corresponds to photonic vision in combination with an ideal monochromatic light source with a wavelength of $\lambda =555\mathrm{ nm}$ and is per definition $683 \mathrm{lm}/\mathrm{W}$. Both, ${\Phi }_{R}$ and the luminous efficiency function $V$, depend on the wavelength $\lambda$ (in nm) and describe the emitted power per wavelength of the light source and the wavelength sensitivity of the human eye, respectively. Theoretically, the electromagnetic spectrum is continuous, and the correlation between ${\Phi }_{R}$ and ${\Phi }_{P}$ is defined as a weighted, infinite sum in form of an integral (Eq. 3a). In practice, because the number of measurement points are finite and experimental spectra are always obtained in discrete wavelength steps, the integral can be transformed into a sum (Eq. 3b). In principio, if ${\Phi }_{P}$ is available ${\Phi }_{R}$ can be derived from the Eq. (4a) or (4b),

4a $${\Phi }_{R}=\frac{1}{{K}_{m}}\bullet \underset{380 \mathrm{nm}}{\overset{800 \mathrm{nm}}{\int }}\frac{{\Phi }_{P}\left(\lambda \right)}{V\left(\lambda \right)}d\lambda$$

4b $${\Phi }_{R}=\frac{1}{{K}_{m}}\bullet \sum_{380 \mathrm{nm}}^{800 \mathrm{nm}}\frac{{\Phi }_{P}\left(\lambda \right)}{V\left(\lambda \right)},$$

where ${\Phi }_{P}\left(\lambda \right)$ is the spectral luminous power distribution.

**Fig. 3:**
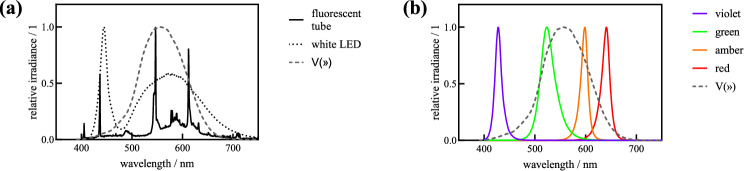
Spectral power distribution of investigated light sources. **a** The relative spectral power distribution of a white fluorescent tube and of a white LED in comparison with the luminous efficiency function of the human eye. Even though both light sources emit white light, their spectral composition differs and show different overlap with the luminous efficiency curve resulting in different luminous flux/radiant flux or photon flux density and therefore can cause a different impact on a biological system. **b** Relative spectral power distribution for common single colored LEDs in the visible spectral range. Depending on the central wavelength and the spectral width of the LED, the overlap with the luminous efficiency curve varies considerably. Violet or red LEDs have a distinct less overlap compared to a green or amber LED leading to larger variations in luminous flux/radiant flux or photon flux density

Depending on the involved measurement devices or the given manufacturer datasheets, radiometric or photometric quantities are given. As a comparison between these systems is tricky and to highlight the different outcome for seemingly similar light condition, the conversion from ${\Phi }_{R}$ into ${\Phi }_{P}$—and vice versa—based on the measured spectral power distributions of a cold white fluorescent lamp, a neutral white LED and four single colored LEDs is shown in Table 3. To simplify comparison, a nominal radiant flux ${\Phi }_{R,nom}$ of 1 W and a nominal luminous flux ${\Phi }_{P,nom}$ of  1000 lm was assumed. The present spectral power distributions and their overlap with the luminous efficiency function is show in Fig. 3.

**Table 3:** Comparison of the radiant flux ${(\Phi }_{R})$ and luminous flux ${(\Phi }_{P})$ for different light sources.

light source	${\Phi }_{P,calc}$ at ${\Phi }_{R,nom}=1 \mathrm{W}$	${\Phi }_{,Rcalc}$ at ${\Phi }_{P,nom}=1000 \mathrm{lm}$
white fluorescent tube	$405 \mathrm{lm}$	$2.47 \mathrm{W}$
white LED	$341 \mathrm{lm}$	$2.93 \mathrm{W}$
violet LED,${\lambda }_{c}=425 \mathrm{nm}$	$45 \mathrm{lm}$	$22.2 \mathrm{W}$
green LED,${\lambda }_{c}=530 \mathrm{nm}$	$466 \mathrm{lm}$	$2.15 \mathrm{W}$
amber LED,${\lambda }_{c}=600 \mathrm{nm}$	$424 \mathrm{lm}$	$2.36 \mathrm{W}$
red LED,${\lambda }_{c}=630 \mathrm{nm}$	$164 \mathrm{lm}$	$6.12 \mathrm{W}$

The calculated luminous flux ${(\Phi }_{Pcalc}),$ based on a nominal radiant flux of ${(\Phi }_{Rnom)}= 1 \mathrm{W},$ was obtained using Eq. 3b and the respective spectral power distribution. The calculated radiant flux ${(\Phi }_{R})$, based on a nominal luminous flux (${\Phi }_{Pnom})=1000 \mathrm{lm},$ was obtained using Eq. 3b and the respective spectral power distribution. Measured spectral power distribution and the overlap with the luminous efficiency function for bright daylight conditions ($V\left(\lambda \right)$) of the discussed light sources are shown in Fig. 3

Due to the luminous efficiency function, the spectral power distribution has a large impact on the resulting luminous flux from a given radiant flux and vice versa. As in Table 3 shown, from a given radiant flux of $1\mathrm{ W}$, the luminous flux varies about one magnitude, from $45\mathrm{ lm}$ for a violet LED (${\lambda }_{C}=425 \mathrm{nm}$) to $466\mathrm{ lm}$ for a green LED (${\lambda }_{C}=530 \mathrm{nm}$). The same is true for a given luminous flux of $1000\mathrm{ lm}$. In this case, the radiant flux varies between $2.15\mathrm{ W}$ and $22.2\mathrm{ W}$. These deviations can be qualitatively understood by overlapping the spectral power distribution of the light source with the luminous efficiency function (see Fig. 3). Taking a white light sources as reference, the resulting luminous flux or radiant flux for sources with a large overlap, like green or amber LEDs, is in the same order. Sources with less overlap, like violet or red LEDs, the resulting flux shows a wider variance. As a rule of thumb, for sources where the maximum in the spectral power distribution is far away from the maximum of the luminous efficiency function the deviations become larger. This is also valid when comparing a white LED and the fluorescence tube, as the white LED has its spectral maximum around $450\mathrm{ nm}$ and therefore its overlap with the luminous efficiency function is less.

These possible deviations are crucial when comparing different light sources with the same luminous flux or radiant flux. To demonstrate this, two extreme cases, a green LED (${\lambda }_{C}=530 \mathrm{nm}$) and a red LED (${\lambda }_{C}=630 \mathrm{nm}$) with the same luminous flux of ${\Phi }_{P}=1000\mathrm{ lm}$ will be closer examined. As Table 3 shows, the resulting radiant flux differs by a factor of almost three, as the fluxes are ${\Phi }_{R}=2.15 \mathrm{W}$ for the green source and ${\Phi }_{R}=6.12 \mathrm{W}$ for the red source. This is also true for a given radiant flux. Comparing a green LED and a red LED with the same radiant flux of ${\Phi }_{R}=1\mathrm{ W}$ from Table 3, the present luminous flux is ${\Phi }_{P}=466 \mathrm{lm}$ for the green light and ${\Phi }_{P}=164 \mathrm{lm}$ for the red light. If light data concerning flux or intensities are present in photometric and radiometric systems and have to be compared, a conversion, which includes the spectral power distribution of the involved sources, into the same system is necessary to draw correct conclusions.

### Comparing light sources in photon-based systems

Photon-based systems take the number of emitted photons per seconds into account, which is proportional to the total emitted energy per second of the certain light source. This can be measured as the total radiant flux ($\Phi )$. Correlating the radiant flux $\Phi$ to a specific area $A$, the respective quantity is irradiance. Starting from Eq. 2 and from a given irradiance at a certain wavelength ${E}_{R,\lambda }$, the photon flux density $PF{D}_{\lambda }$ at a certain wavelength $\lambda$ in $\mathrm{mol }{\mathrm{s}}^{-1} {\mathrm{m}}^{-2}$ can be calculated:

5 $$PF{D}_{\lambda }=\frac{\frac{{\Phi }_{R,\lambda }}{A}}{{Q}_{\mathrm{photon}}\bullet {N}_{A}}=\frac{{E}_{R,\lambda }}{{Q}_{\mathrm{photon}}\bullet {N}_{A}}=\frac{{E}_{R,\lambda }\bullet \lambda }{c\bullet h\bullet {N}_{A}}$$

With a given spectral intensity distribution ${E}_{R}\left(\lambda \right)$, the total $PFD$ in $\mu \mathrm{mol }{\mathrm{s}}^{-1} {\mathrm{m}}^{-2}$ can be derived from Eq. 5 as a sum over all wavelengths^1^

6 $$PFD={10}^{6}\bullet \sum_{{i=\lambda }_{1}}^{{\lambda }_{2}}PFD\left({\lambda }_{i}\right)=\frac{1{0}^{6}}{c\bullet h\bullet {N}_{A}}\bullet \sum_{{i=\lambda }_{1}}^{{\lambda }_{2}}{E}_{R}\left({\lambda }_{i}\right)\bullet {\lambda }_{i},$$

where ${\lambda }_{1}$ is the lower wavelength bound and ${\lambda }_{2}$ is the upper wavelength bound of the relevant spectrum, depending on the BWF. For PAR these boundaries corresponds to ${\lambda }_{1}=400 \mathrm{nm}$ and to ${\lambda }_{2}=700 \mathrm{nm}$. If the $PFD$ cannot measured directly with spectral radiometer, the $PFD$ can be derived from Eq. 6 using irradiance data from a common radiometer and the spectral power distribution measured with a spectrometer or supplied by the manufacturer`s data sheets. Calculated $PF{D}_{calc}$ of four light sources—previously described—are shown in Table 4. To simplify comparison, the irradiance of all light sources was assumed ${E}_{R}=1 \mathrm{W }{\mathrm{m}}^{-2}$ and ${E}_{P}=1000\mathrm{ lx}$. As in Table 4 shown, the resulting photon flux densities varies for different light sources between $3.7 \mathrm{\mu mol }{\mathrm{s}}^{-1} {\mathrm{m}}^{-2}$ for a violet LED and $5.3 \mathrm{\mu mol }{\mathrm{s}}^{-1} {\mathrm{m}}^{-2}$ for a red LED even though the same irradiance was assumed. This is due to their different spectral power distribution (see Fig. 3). Determining the $PFD$ for light sources that are characterized by photometric quantities, the spectral dependance will result in much larger deviations. From a given illuminance of ${E}_{P}=1000 \mathrm{lx}$, the resulting photon flux densities varies from $PF{D}_{calc, green}=9.5 \mathrm{\mu mol }{\mathrm{s}}^{-1} {\mathrm{m}}^{-2}$ for the green light source to $PF{D}_{calc, red}=32 \mathrm{\mu mol }{\mathrm{s}}^{-1} {\mathrm{m}}^{-2}$ for the red source to $PF{D}_{calc, violet}=82 \mathrm{\mu mol }{\mathrm{s}}^{-1} {\mathrm{m}}^{-2}$. The resulting photon flux densities for white light sources are $PF{D}_{calc, fluorescent tube}=12 \mathrm{\mu mol }{\mathrm{s}}^{-1} {\mathrm{m}}^{-2}$ and $PF{D}_{calc, white LED}=14 \mathrm{\mu mol }{\mathrm{s}}^{-1} {\mathrm{m}}^{-2}$. $PFD$ value for a fluorescent tubes is equal to the calculated with $PF{D}_{literature, fluorescent tube}=12 \mathrm{\mu mol }{\mathrm{s}}^{-1} {\mathrm{m}}^{-2}$ (McCree 1972). The values for white light sources are in the same range as green or amber light sources around $10 \mathrm{\mu mol }{\mathrm{s}}^{-1} {\mathrm{m}}^{-2}$, which is not surprising as the white light and the green and amber light have closer central wavelengths as red and violet light. In addition, the overlapping of the spectral power distribution of these light sources with the luminous efficiency function is larger compared to red or violet light (see Fig. 3). These findings coincide with the resulting radiant or luminous fluxes from Table 3, where the deviations are much larger for red and violet light compared to white, green or amber light.

**Table 4:** Calculated photon flux density $(PF{D}_{calc})$ for different light sources.

light source	$PF{D}_{calc}$ at ${E}_{R}=1 \mathrm{W }{\mathrm{m}}^{-2}$	$PF{D}_{calc}$ at ${E}_{P}=1000 \mathrm{lx}$
white fluorescent tube	$4.74 \frac{\mathrm{\mu mol}}{\mathrm{s }{\mathrm{m}}^{2}}$	$11.7 \frac{\mathrm{\mu mol}}{\mathrm{s }{\mathrm{m}}^{2}}$
white LED	$4.61 \frac{\mathrm{\mu mol}}{\mathrm{s }{\mathrm{m}}^{2}}$	$13.5 \frac{\mathrm{\mu mol}}{\mathrm{s }{\mathrm{m}}^{2}}$
violet LED,${\lambda }_{c}=425 \mathrm{nm}$	$3.69 \frac{\mathrm{\mu mol}}{\mathrm{s }{\mathrm{m}}^{2}}$	$81.9 \frac{\mathrm{\mu mol}}{\mathrm{s }{\mathrm{m}}^{2}}$
green LED,${\lambda }_{c}=530 \mathrm{nm}$	$4.44 \frac{\mathrm{\mu mol}}{\mathrm{s }{\mathrm{m}}^{2}}$	$9.53 \frac{\mathrm{\mu mol}}{\mathrm{s }{\mathrm{m}}^{2}}$
amber LED,${\lambda }_{c}=600 \mathrm{nm}$	$4.92 \frac{\mathrm{\mu mol}}{\mathrm{s }{\mathrm{m}}^{2}}$	$11.6 \frac{\mathrm{\mu mol}}{\mathrm{s }{\mathrm{m}}^{2}}$
red LED,${\lambda }_{c}=630 \mathrm{nm}$	$5.26 \frac{\mathrm{\mu mol}}{\mathrm{s }{\mathrm{m}}^{2}}$	$32.2 \frac{\mathrm{\mu mol}}{\mathrm{s }{\mathrm{m}}^{2}}$

Calculation were performed according to Eq. (6) with a nominal irradiance ${E}_{R,nom}=1 \mathrm{W }{\mathrm{m}}^{-2}$ and a nominal illuminance ${E}_{P,nom}=1000 \mathrm{lm}$. Photometric illuminance was converted into radiometric irradiance according Eq. 4b prior $PFD$ calculation. The corresponding spectral power distributions are shown in Fig. 3

Our results regarding photon flux densities for different light sources with the same nominal irradiance or illuminance, highlight that a straightforward comparison is not possible and may lead to incorrect conclusions. Therefore, it is important to determine the present photon flux densities considering the spectral power distribution of the involved light source.

### A rule of thumb to convert light quantities without exact spectral data

Most manufacturer data, like luminous flux ${\Phi }_{P}$, radiant flux ${\Phi }_{R}$ or luminous intensity ${I}_{ p}$—are based on different systems, thus, making a comparison or interpretation of these datasets not trivial. To verify manufacturer`s specification through measurements may be tricky as well, if no suitable equipment is available. However, as proposed in the next section, a transformation in-between these quantities without knowing the spectral properties of the used light source is partly possible, if conversion factors are taken into consideration.

#### Approximations in energy-based systems

For a known spectral power distribution, both, ${\Phi }_{R}$ and ${\Phi }_{P}$, can be calculated according to Eqs. 3b or 4b, respectively. In case no spectral information are available, the introduction of conversion factors (${\kappa }_{{\lambda }_{c}}$) could be expedient. In contrast to conventional light sources such as cold white fluorescent lamps with a ${\kappa }_{{\lambda }_{c}}$ of $2.71 \mathrm{mW }{\mathrm{lm}}^{-1}$ or high pressure sodium lamp with a ${\kappa }_{{\lambda }_{c}}$ of $3.01 \mathrm{mW }{\mathrm{lm}}^{-1}$ (McCree 1972), conversion factors for LEDs are rather difficult to estimate, due to the variety of different LED types. However, a rough approximation can be assessed by extracting the ${\kappa }_{{\lambda }_{c}}$ from the luminous efficiency function and the specified peak or dominant wavelength (= central wavelength;${\lambda }_{c}$), obtained from the manufacturer’s datasheet. In the proposed conversation factors from Table 5, ${\kappa }_{{\lambda }_{c}}$ corresponds to the average value of the luminous efficiency function around the central wavelength. The luminous function values were taken from (Stockman and Sharpe 2000) and averaging calculation was done for a spectral width of 20 nm, which reflects typical spectral distributions of single coloured LEDs. Conversion factors ${\kappa }_{{\lambda }_{c}}$ based on common central wavelengths are summarized in Table 5. ${\Phi }_{P, calc}$ (in lumen) and ${\Phi }_{R,calc}$ (in watt) can be calculated using the Eqs. 3b and 4b. The maximal luminous efficacy ${K}_{m}=683\frac{\mathrm{lm}}{\mathrm{W}}$. Table 6 compares these fluxes, with ${\Phi }_{R,approx}$ and ${\Phi }_{P,approx}$ derived from Eqs. 7a and 7b using the proposed conversion factors ${\kappa }_{{\lambda }_{c}}$ from Table 5.

**Table 5:** Conversion factors $({\kappa }_{{\lambda }_{c}})$ based on common central wavelengths ${(\lambda }_{c})$ in the range of 425 to 665 nm.

central wavelength ${\lambda }_{c}$/nm	conversion factor ${\kappa }_{{\lambda }_{c}}$/1	central wavelength ${\lambda }_{c}$/nm	conversion factor ${\kappa }_{{\lambda }_{c}}$/1
425	0.02	530	0.85
450	0.07	600	0.69
470	0.13	630	0.30
500	0.36	665	0.03

The factors are an average value taken from the luminous efficiency function around a known ${\lambda }_{c}$. For single coloured LEDs a typical spectral full width at half maximum value of $20 \mathrm{nm}$ was assumed as average range. ${\lambda }_{ c}$ are based on the datasheets of Lumileds Luxeon CZ colour line

**Table 6:** Comparison of fluxes (${\Phi }_{R,calc}, {\Phi }_{P,calc}$) calculated by Eq. 3b and 4b and “approximated” fluxes (${\Phi }_{R,approx}, {\Phi }_{P,approx}$) calculated by Eq. 7a, 7b for five common central LED-wavelengths.

central LED wavelength	${\Phi }_{P}\mapsto {\Phi }_{R}$ for ${\Phi }_{P}=1000 \mathrm{lm}$		${\Phi }_{R}\mapsto {\Phi }_{P}$ for ${\Phi }_{R}=1 \mathrm{W}$	
${\lambda }_{c}$/ nm	${\Phi }_{R, calc}$/W	${\Phi }_{R, approx}$/W	${\Phi }_{P, calc}$/lm	${\Phi }_{P, approx}$/lm
425 (violet LED)	22	72	45	14
470 (blue LED)	6.7	11	151	89
530 (green LED)	2.2	1.7	466	580
600 (amber LED)	2.4	2.1	424	471
630 (red LED)	6.1	4.8	164	206

The conversion factors were taken from Table 5. For the conversion from a photometric system to a radiometric system, a starting luminous flux of ${\Phi }_{P}=1000 \mathrm{lm}$ was assumed. For the inverse conversion from a radiometric system to photometric, the starting radiant flux was ${\Phi }_{R}=1 \mathrm{W}$. Fluxed were calculated as shown in Table 3

7a $${\Phi }_{P,approx}={\Phi }_{R}{\bullet {K}_{m}\bullet \kappa }_{{\lambda }_{c}},$$

7b $${\Phi }_{R,approx}=\frac{{\Phi }_{P}}{{K}_{m}{\bullet \kappa }_{{\lambda }_{c}}},$$

As shown in Table 6, calculations based on the Eq. 7a and 7b were found partially effective. For LEDs with a central wavelength close to the maximum of the luminous efficiency function—around $555\mathrm{ nm}$—calculations and approximations were found in line, e.g. for a green LED (${\lambda }_{c}= 530\mathrm{ nm}$) ${\Phi }_{R, calc}=2.2 \mathrm{W}$ and ${\Phi }_{R, approx}=1.8 \mathrm{W}$ could be revealed. In contrast, for LEDs with a central wavelength further away from 555 nm, the deviations between calculated and “approximated” results were found distinct, e.g. ${\Phi }_{R, calc}=22 \mathrm{W}$ versus ${\Phi }_{R, approx}=70 \mathrm{W}$ for violet LED (${\lambda }_{c}= 425\mathrm{ nm}$). These deviations may be explained by the shape of the luminous efficiency function, e. g. revealing values around 425 nm close to zero and a flat gradient, as well as less overlapping between the spectral power distribution and the luminous efficiency function, as discussed before. Similar findings could be expected for deep-red LEDs with wavelengths around $680\mathrm{ nm}$. Nevertheless, the here proposed equations allows a “rough approximation” of the present order of magnitude and can be used for a first indication. To interpret light quantities in more details, an exact conversion according to Eqs. 3b or 4b is mandatory.

#### “Approximations” in photon-based systems

If spectral information of the light source are not specified, an exact determination of the photon flux quantities is not possible. However, the $PFD$ can be “approximated” by using conversion factors: e.g. for cold white fluorescent lamps a ${\kappa }_{{\lambda }_{c}}$ of $4.59 \mathrm{\mu mol }{\mathrm{s}}^{-1}{\mathrm{m}}^{-2}\mathrm{ per W }{\mathrm{m}}^{-2}$ or for high pressure sodium lamps a ${\kappa }_{{\lambda }_{c}}$ of $4.98 \mathrm{\mu mol }{\mathrm{s}}^{-1}{\mathrm{m}}^{-2}\mathrm{ per W }{\mathrm{m}}^{-2}$ can be used (Thimijan and Heins 1983). To our best knowledge (Meyer-Arendt 1968; McCree 1971, 1972, 1973, 1981), most conversion factors described in literature are based on conventional light sources, but are rarely described for LEDs, which cover a wide range of colours. However, for the latter, the $PFD$ can be “approximated” from the central wavelength ${\lambda }_{c}$, using Eq. 8. Photon flux densities for different LED light sources with a nominal irradiance of ${E}_{R}=1 \mathrm{W }{\mathrm{m}}^{-2}$ were calculated with Eq. 6 ($PF{D}_{calc}$) and compared to results of Eq. 8 ($PF{D}_{approx}$) in Table 7.

**Table 7:** Comparison of calculated photon flux densities $(PF{D}_{calc}$) from Eq. 6 and “approximated” photon flux densities $(PF{D}_{approx}$) using Eq. 8 for four single coloured LEDs and two white light sources

central wavelength	${E}_{R}=1 \mathrm{W }{\mathrm{m}}^{-2}$	
${\lambda }_{c}$/ nm	$PF{D}_{calc}$/$\mathrm{\mu mol }{\mathrm{s}}^{-1} {\mathrm{m}}^{-2}$	$PF{D}_{approx}$/$\mathrm{\mu mol }{\mathrm{s}}^{-1} {\mathrm{m}}^{-2}$
425 (violet LED)	3.7	3.6
530 (green LED)	4.4	4.4
550 (white, fluorescent tube)	4.7	4.6
550 (white, LED)	4.6	4.6
600 (amber LED)	4.9	5.0
630 (red LED)	5.3	5.4

For both white light sources, a central wavelength of ${\lambda }_{c}=550 \mathrm{nm}$ was assumed. For the single coloured LEDs, the peak wavelength was set the central wavelength

8 $$PF{D}_{approx}=\frac{{E}_{R}\bullet {\lambda }_{c}}{c\bullet h\bullet {N}_{A}}$$

Both, $PF{D}_{calc}$ and $PF{D}_{approx}$, revealed densities with a deviation below 4%. For the white fluorescence tube, results of $PF{D}_{calc} (4.7 \mathrm{\mu mol }{\mathrm{s}}^{-1} {\mathrm{m}}^{-2}$) and $PF{D}_{approx} (4.6 \mathrm{\mu mol }{\mathrm{s}}^{-1} {\mathrm{m}}^{-2 })$ were found in line with a literate-based $PFD$ of $4.7 \mathrm{\mu mol }{\mathrm{s}}^{-1} {\mathrm{m}}^{-2}$ (McCree 1972).

In case the illuminance is specified but no exact spectral power distribution is available, $PFDs$ can be “approximated” in a two-step conversion: First, the irradiance can be “approximated” using Eq. 7b and by inserting ${E}_{P}$ rather than ${\Phi }_{P}$. In the second step, the $PFD$ can be “approximated” by the Eq. 8. As there are two sequential “approximation” steps involved, result should be seen as rough estimations, particularly for LEDs with central wavelengths close to the boarder of the visible range of the electromagnetic spectrum: For a green LED (${\lambda }_{c}=530 \mathrm{nm}$,${\Phi }_{R, approx}=1.7\mathrm{ W}$, ${E}_{P}= 1000\mathrm{ lx}$) photon flux densities—$PF{D}_{approx,green}=8 \mathrm{\mu mol }{\mathrm{s}}^{-1} {\mathrm{m}}^{-2}$ and $PF{D}_{calc, green} =9.5 \mathrm{\mu mol }{\mathrm{s}}^{-1} {\mathrm{m}}^{-2}$—were found in line, but distinguished strongly for a violet LED (${\lambda }_{c}=425 \mathrm{nm},{\Phi }_{R, approx}=72\mathrm{ W}$, ${E}_{P}= 1000\mathrm{ lx}$)—$PF{D}_{approx, violet}=257 \mathrm{\mu mol }{\mathrm{s}}^{-1} {\mathrm{m}}^{-2}$ and $PF{D}_{calc, violet}=82 \mathrm{\mu mol }{\mathrm{s}}^{-1} {\mathrm{m}}^{-2}$. This two-step approximation is accompanied by a large error for central wavelengths far away from the maximum of the luminous efficiency function.

Taking these conversions into account is crucial for a straightforward characterization of illumination conditions. The literature research conducted on “lux” or “lx” & “green algae” vs “μmolm − 2 s − 1” & “green algae” (as previously described in 1.) illustrates the inconvenience of using different “terminologies” when it comes to the interrelation between illumination and target organisms. From the perspective of photosynthetic organism, a photometric characterization is incorrect, as the spectral sensitivity of the human eye is not relevant to photosynthesis and photon-based quantities such as $PFD$ in μmolm^−2^ s^−1^ should be used.

### Measuring lighting regimes

Besides a basic understanding of these physical principles, their practical application in illumination experiments is of great importance to generate comparable and valid data sets. This chapter provides a short excursus into common, existing light measurement sensors and their application in studies dealing with phototrophic organisms. A calibrated spectroradiometer, which is capable to provide the full spectral distribution of a light source (Pritchard et al. 2013; Jegan et al. 2022), is a recommended investment to properly characterize illumination regimes. With such devices, photon-based quantities, such as $PFD$ can be directly measured without further conversions. If spectroradiometer are not available, photon-based quantities can be measured with quantum sensors. The measured $PFD$, as described in Sect. "Photon-based system", depends on the spectral power distribution of the light source and the incident irradiance. The different responses of quantum sensors to the spectral power distribution of various light sources should be considered with correction factors. These factors are usually provided by the manufacturer. It is important to stress out, that different light sources need different correction factors, otherwise parts of the emitted wavelengths are over- or under detected. This is especially crucial for single coloured LEDs, which emit at different central wavelengths. If the correction factors are not properly chosen, the measured $PFD$ from quantum sensors is incorrect. Another possibility to determine the $PFD$ is using an optical power sensor to measure the irradiance and calculate the $PFD$ with the spectral power distribution of the light source as described in Sect. "Comparing light sources in photon-based systems" or estimate the $PFD$ according to the proposed approximation in Sect. "Approximations in energy-based systems".

As the irradiance depends on the distance from the light source, the position of the sensor is crucial for the measurement of the light related quantity. The location of the sensor should reflect the present experimental setup, such as the sample plane or the center of the photobioreactor. Regarding functional principle, there mainly exist two types of sensors for measuring lighting regimes: planar or flat sensors and spherical sensors. Planar sensors measure light that impinges on their surface from a downward direction. Hence, the angle between the sensor and target light sources could have a significant effect on the sensors’ response and thus should be adjusted with great care and stated in the publication (Long et al. 2012). A sensor with a cosine correction can be used to compensate for the dependence of the measured power from the incident angle to a certain extent (Loogman and van Liere 1978). For plant research, where for example a plant leaf can be approximated as planar, flat sensors are appropriate. However, for experimental setups, where light comes from any direction, spherical sensors should be used (Björn 2015). Such sensors have a spherical or hemispherical collecting surface and are capable of detecting light from $2-4\pi \mathrm{sr}$ (Long et al. 2012). For complex three-dimensional biological systems, underwater applications or submerged cultivation of microalgae in photobioreactors, many authors have suggested to use spherical systems, because these sensors are capable to additionally detect the scattering and attenuation of light due to interactions with particles, molecules, and surrounding objects that could have affect the experiments (Arst et al. 2000; Long et al. 2012). There exist publications that provide theory and technical information to develop custom sensing solutions (e. g. Jegan et al. 2022). Jegan et al. (2022) also published a list of options for those seeking to use commercially available sensor systems.

## Summary and conclusion

As shown in this study, multiple equations and assumptions allow a straightforward conversions within and in-between the energy- and photon-based system, even for a weak database. However, for data interpretation, it has to been taken into consideration that different light sources with the same ${\Phi }_{R}$/${\Phi }_{P}$ and subsequently the same ${E}_{R}$/${E}_{P}$ or the same ${I}_{R}$/${I}_{p}$ may not have the same biological impact. For example, a key quantity to describe biological processes related to photosynthesis is the $PFD$, which describes the number of photons arriving in a certain time on a certain area. As the number of photons depends on the emitted power and the involved wavelengths, two different light sources with the same radiant or luminous flux will not result in the same $PFD$, thus the biological impact on the target organism will differ significantly. Hence, when comparing light properties, a conversion of all quantities into the same system, preferable into photon-based quantities, would be expedient to avoid wrong interpretations and conclusions. LED-based technology holds the key to the future revolution for manifold life-science applications; however a fundamental knowledge of users is crucial in order to push ahead with the technical LED-innovations. Researchers have to be aware of the many different existing light related quantities. Therefore we call attention to the following points, when working with light or characterizing illumination conditions: (1) Seemingly similar light sources regarding optical output may vary in the biological impact due to their different spectral power distribution. To verify, use a calibrated spectroradiometer, equipped with a proper sensor system. (2) If the involved biological response is not common knowledge or the biological system consists of a multitude of organisms, it is crucial to use unweighted quantities in the energy- or photon-based system. (3) Photometric based quantities and the corresponding units such as illuminance in lux or the luminous flux in lumen are not suitable to characterize photosynthetic organism and should not be used. (4) Only apply biological weighting functions when the spectral response of the biological system is known without doubt. (5) When using quantum sensors without the capability to measure spectral information, proper correction factors must be used. This is especially crucial for single coloured light sources such as LEDs. (6) If quantum sensors are not available, the $PFD$ should be calculated with the spectral data from the incident light and the measured irradiance from an optical power sensor as described in this work. Furthermore, for single coloured light sources, the $PFD$ can be estimated in good approximation with conversion factors proposed in this work.

## Data Availability

Data will be made available on reasonable request. The datasets presented in this study can be found in online repositories. The names of the repository/repositories and accession number(s) can be found in the article.

## Footnotes

1 The electromagnetic spectrum is continuous. Therefore the correct calculation would be in form of an integral equation $PFD=\underset{{\lambda }_{1}}{\overset{{\lambda }_{2}}{\int }}PFD\left(\lambda \right)d\lambda$. Since experimental spectra are always obtained in discrete wavelength steps, the calculation by summation is valid.
